# Different activity and toxicity of immunotherapy in monozygotic twins diagnosed with early triple-negative breast cancer: a case report

**DOI:** 10.1177/17588359241297565

**Published:** 2025-02-18

**Authors:** Marta Perachino, Lucia Del Mastro, Giulia Buzzatti, Lucia Trevisan, Maria Grazia Razeti, Andrea Bellodi, Stefano Spinaci, Davide Soldato, Matteo Lambertini, Francesca Poggio

**Affiliations:** Department of Medical Oncology, U.O. Clinica di Oncologia Medica, IRCCS Ospedale Policlinico San Martino, Genoa, Italy; Department of Medical Oncology, U.O. Clinica di Oncologia Medica, IRCCS Ospedale Policlinico San Martino, Genoa, Italy; Department of Internal Medicine and Medical Specialties, School of Medicine, University of Genova, Genoa, Italy; UO Oncologia Medica 2, IRCCS Ospedale Policlinico San Martino, Genoa, Italy; Hereditary Cancer Unit, IRCCS Ospedale Policlinico San Martino, Genoa, Italy; Hereditary Cancer Unit, IRCCS Ospedale Policlinico San Martino, Genoa, Italy; Department of Medical Oncology, U.O. Clinica di Oncologia Medica, IRCCS Ospedale Policlinico San Martino, Genoa, Italy; Department of Internal Medicine and Medical Specialties, School of Medicine, University of Genova, Genoa, Italy; Division of Breast Surgery, Ospedale Villa Scassi and ASL3, Genoa, Italy; Department of Medical Oncology, U.O. Clinica di Oncologia Medica, IRCCS Ospedale Policlinico San Martino, Genoa, Italy; Department of Medical Oncology, U.O. Clinica di Oncologia Medica, IRCCS Ospedale Policlinico San Martino, Genoa, Italy; Department of Internal Medicine and Medical Specialties, School of Medicine, University of Genova, Genoa, Italy; Department of Medical Oncology, U.O. Clinica di Oncologia Medica, IRCCS Ospedale Policlinico San Martino, Largo Rosanna Benzi, 10, Genova 16132, Italy

**Keywords:** breast cancer, case report, immunotherapy, toxicity

## Abstract

Breast cancer (BC) is the most common malignancy among women. Among 5%–10% of diagnoses are correlated with hereditary cancer syndromes, while the remaining cases are sporadic and linked to multiple factors. When a pathogenetic variant in one of the genes commonly associated with BC is detected, the patient is referred to a tailored surveillance program; otherwise, the standard follow-up guidelines are applied. We present a unique case of BC diagnosed in two monozygotic twins at the same age apparently unrelated to a hereditary syndrome known to date. Notably, despite comparable clinical–pathological features, the two neoplasms behaved differently to neoadjuvant chemo-immunotherapy, showing different outcomes and toxicities. Very little is known about the predictive mechanisms of response and toxicity to immunotherapy and this clinical case might be a starting point for further investigations.

## Introduction

Breast cancer (BC) is the most frequent neoplasia among women.^1^ In addition to various modifiable risk factors such as smoking, estrogen exposure, low parity, obesity, and alcohol consumption, genetics also play a significant role.^2^

*BRCA1* was the first high-penetrance gene identified as being associated with hereditary BC. Together with *BRCA2*, these genes are estimated to account for nearly 15% of familial BC cases.^2^ Currently, other genes associated with hereditary breast/ovarian syndrome have been identified, including those with high penetrance (*PALB2, CDH1, STK11, PTEN*, and *TP53*) and moderate penetrance (*ATM, BARD1, CHEK2, RAD51C*, and *RAD51D*).^3–11^

Family history is a well-known factor with a major impact on the risk of developing BC; however, not all patients with a family history of BC carry common or rare mutations. This may be because multiple factors linked to BC development can occur. For instance, breast density and body mass index also tend to cluster within families, likely due to both genetic inheritance and shared lifestyles. These findings suggest that a more detailed evaluation of family history, along with environmental evaluation, could have significant clinical implications; therefore, they need to be investigated.

Twins provide a natural study population for genetic epidemiology, particularly when they are genetically identical. If heritable factors contribute to the development of a disease, concordance rates should be higher in monozygotic twins compared to in dizygotic twins. In the literature, cases of BC arising in twins have been reported, most commonly associated with *BRCA1/2* variants.^8–10^ The incidence of BC in monozygotic twins is four times higher than in the mothers and sisters of patients, especially after age 55, with a lifetime cumulative risk of BC for identical twins estimated at 20%–30%.^11^ A simple Mendelian genetic model, however, does not fully account for this elevated risk, as it predicts much lower rates of BC in this population. While genetic susceptibility plays a role—increasing the risk with an affected relative—shared environmental factors, such as a shared placenta, may also significantly contribute to the high disease concordance in monozygotic twins.^11^ On the other hand, the disease discordance observed in some identical twin pairs may point to external influences. Indeed, smaller studies on monozygotic twins have demonstrated high discordance, which has been attributed to environmental factors.^9^ Epigenetic factors, influenced by environmental elements such as nutrition, toxicants, and ethanol, may also play a role, with DNA methylation being a key example. In sporadic cancers, epigenetic gene silencing through *BRCA1* hypermethylation has been observed, suggesting its potential involvement in tumorigenesis. There is also some evidence that this mechanism could help explain BC discordance in monozygotic twin pairs.^12^

We have encountered a unique case that involves two monozygotic twins diagnosed with triple-negative early BC (TNBC) at the same age, without any apparent genetic predisposition. They exhibited a different response to oncological treatments in terms of both efficacy and toxicity.

## Case presentation

The reporting of this study conforms to the CARE checklist^13^ (Supplemental Material).

### Diagnosis

**Figure 1.**
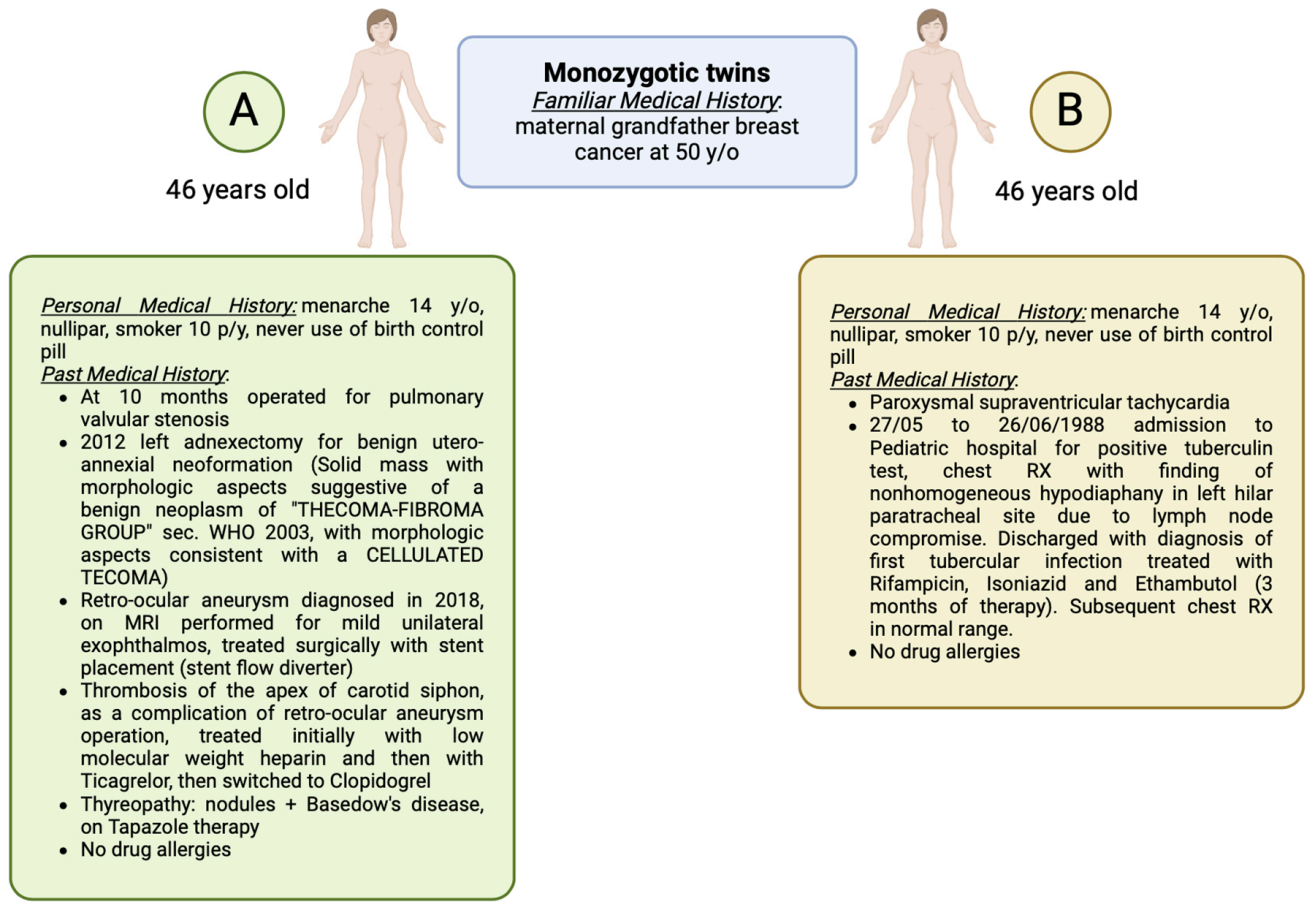
Medical history of A and B.

The first twin (Twin A) came to our attention at the age of 46 years due to a self-detected lump on her left breast in June 2022. Her past medical history is summarized in Figure 1.

Her mammogram (in June 2022) revealed a 31 mm nodule in the outer upper quadrant (OUQ) of the left breast, without any suspicious lymphadenopathies. On the 16th of June, she underwent an ultrasound-guided core biopsy confirming an invasive ductal carcinoma of the breast with negative expression of hormone receptors (Estrogen Receptor (ER) 0% Progesteron Receptor (PgR) 0%), high proliferation index (Ki67 70%–80%), HER2 score 1+ with focal 2+ in 10%–15% of neoplastic cells (fluorescence in situ hybridization—not amplified).

**Figure 2.**
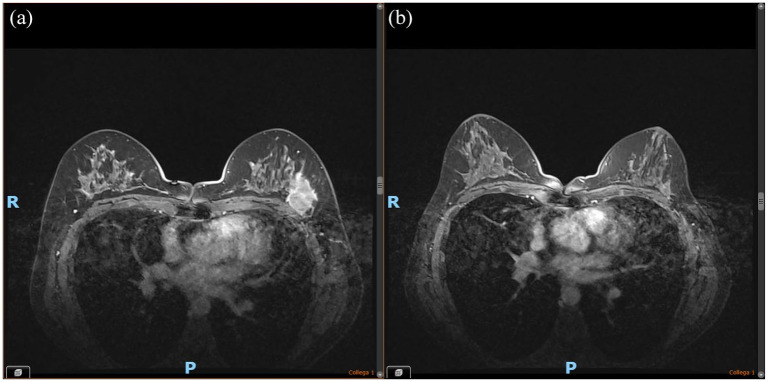
Breast MRI of Twin A. (a) Baseline. (b) Post-neoadjuvant therapy.

At the clinical visit, a 4 cm nodule was palpable on the OUQ of the left breast with no evidence of ipsilateral lymphadenopathies. The diagnostic work-up (total body CT scan) was negative for metastatic dissemination and the breast MRI (Figure 2(a)) confirmed a single nodule and suspicious ipsilateral lymphadenopathy (the cytologic sample tested positive for neoplastic cells). Furthermore, a charcoal mark on both the breast and lymph node was applied for potential subsequent conservative surgery.

Given the TNBC phenotype, the patient was referred to genetic counseling per guidelines.^1^ Extended genetic testing was performed and no pathogenetic variants were detected in the analyzed genes (*BRCA1, BRCA2, ATM, BARD1, BRIP1, CDH1, CHEK2, PALB2, RAD51C, RAD51D*, and *TP53*).

One month later, in July 2022, her monozygotic twin (Twin B) came to our attention reporting a mammogram with evidence of suspicious microcalcifications in the context of a thickening in the left breast. An echotomography was done, showing a hypoechoic nodular lesion with spiculated margins of 1.7 cm on the left OUQ, plus a suspicious ipsilateral lymphadenopathy of 1.7 cm. The core biopsy showed an invasive ductal carcinoma of the breast with TNBC phenotype (ER 0% PgR 0% HER2 score 0, Ki67 30%–35%) with metastasis in the ipsilateral axillary lymph node.

Her past medical history is summarized in Figure 1. Twin A and Twin B’s maternal grandfather was diagnosed with BC at the age of 50, he was not tested for *BRCA* mutations; no other evidence of oncological familiarity was reported.

**Figure 3.**
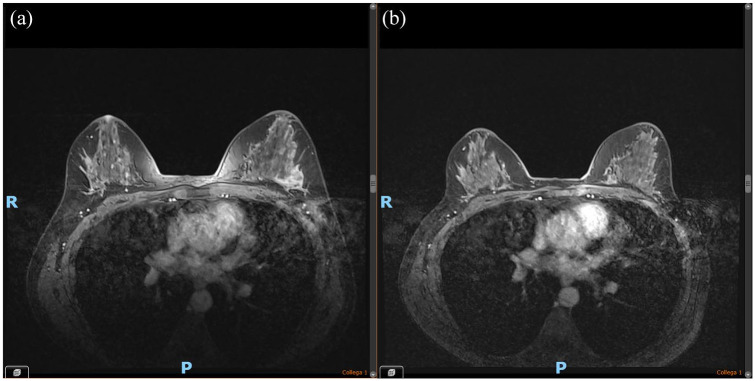
Breast MRI of Twin B. (a) Baseline. (b) Post-neoadjuvant therapy.

Total body CT scan plus breast MRI of Twin B did not show metastatic dissemination while confirming the presence of a single left breast nodule plus one ipsilateral metastatic lymphadenopathy (Figure 3(a)).

Twin B was referred to our genetic clinic where *BRCA1/2* testing did not reveal any pathogenic variants. In addition, the patient independently underwent whole-exome sequencing (WES) at a laboratory outside our Institution.

### Genetic analysis and WES results

Twin A and Twin B underwent genetic testing at our Institution.

Both library preparation and chip loading were automatically performed on the Ion Chef System and sequencing was performed by next-generation sequencing (NGS) on the S5 Ion Torrent platform with a custom Ion ample seq on-demand Thermofisher panel with an average read depth >100×. The bioinformatic analysis was performed on the S5 Torrent Server VM using Coverage Analysis and Variant Caller plugins and on Ion Reporter software; bam-files were uploaded to Alamut software. Pathogenic, probably pathogenic variants, and variants of uncertain significance identified by NGS were confirmed by Sanger sequencing. MLPA was performed on DNA extracted from peripheral blood using SALSA MLPA Kit MRC-Holland analysis software Coffalyser.net. For the classification of variants identified in the *CDH1* gene, the InSight database was taken as a reference (https://www.insight-database.org/); while for *TP53* variants, the database http://p53.iarc.fr is taken as a reference as Brcaexchange database (https://brcaexchange.org/) and ENIGMA criteria (http://www.enigmaconsortium.org/) for *BRCA1/2* variants.

A diagnostic test based on WES was employed for Twin B in which the analysis was limited to variants detected in the specific gene or genes possibly related to the patient’s clinical indications.

Extracted genomic DNA was processed using the ExomeCG—CytoGenomics capture assay kit (Nonacus, Birmingham, United Kingdom) and sequenced on an NGS sequencer (Illumina, San Diego, California, U.S.) with an average coverage of 100×. Sequencing data were processed using the bioinformatics pipeline developed for this specific use. Mapping and analysis were based on the UCSC hg 19 reference sequence of the human genome. The filters used to flag variants in the analyzed genes were of minor allele frequency (MAF) <1% (AF max <0.01).

The entire exome analysis was performed according to the recommendations of the American College of Medical Genetics and Genomics guidelines.^14^ Pathogenic, probably pathogenic, and Variants of Uncertain Significance (VUS) were included in the report, while benign and probably benign variants were not reported. The following genes have been tested: *AIP, ALK, APC, ATM, ATR, AXIN2, BAP1, BARD1, BLM, BMPR1A, BRCA1, BRCA2, BRIP1, BUB1B, CASR, CDC73, CDH1, CDK4, CDKN1B, CDKN1C, CDKNZA, CEBPA, CHEK2, CTC1, CTNNA1, CYLD, DDB2, DICER1, DIS3L2, DKC1, EGLN1, EPCAM, ERGC1, ERCC2, ERCC3, ERCCA, ERCC5, EXT1, EXT2, EZH2, FAN1, FANCA, FANCB, FANCC, FANCD2, FANCE, FANCF, FANGG, FANGI, FANGL, FANCM, FH, FLCN, GALNT12, GATA2, GPC3, GREMI, HOXB13, HRAS, KIF1B, KIT, LZTR1, MAX, MC1R, MENT, MET, MITE, MLH1, MLH3, MRE11, MSH2, MSH6, MUTYH, NBN, NF1, NF2, NHP2, NOP10, NTHL1, PALB2, PDGFRA, PHOX2B, PMS2, POLD1, POLE, POLH, POT1, PRKARIA, PRSS1, PTCH1, PTCH2, PTEN, RAD50, RAD51C, RAD51D, RB1, RECOLA* (*9979*), *RET, RUNX1, SDHA, SDHAF2, SDHB, SDHC, SDHD, SLC45A2, SLX4, SMADA, SMARCA4, SMARCB1, SMARCE1, STK11, SUFU, TERC, TERT, TINF2, MEMI27, TP53, TSC1, TSC2, TYR, VHL, WRAP53, WRN, WT1, XPA, XPC*, and *XRCC2*. After WES and analysis for diagnostic purposes for the clinical reference frame, no single nucleotide variants (SNVs), small indels, or copy number variants (CNVs) were detected in those genes; thus, no definitive molecular cause was identified for the clinical findings in this patient.

### Treatment

In consideration of the clinical stage at diagnosis, both twins underwent neoadjuvant chemotherapy with 12 cycles of weekly paclitaxel 80 mg/mq + carboplatin Area Under the Curve (AUC) 1.5, followed by 4 cycles of epirubicin 90 mg/mq + cyclophosphamide 600 mg/mq (EC); Pembrolizumab (200 mg every 3 weeks) was added within an Expanded Access Programme. EC was administered every 3 weeks, as per protocol.^15,16^

From the third cycle of EC, Twin A began to complain of diarrhea G1 (up to 4 liquid stools per day), treated with methylprednisolone 1 mg/kg/die with benefit. Pembrolizumab treatment was continued as per guidelines.^17^ Post-neoadjuvant breast MRI showed a partial radiologic response (Figure 2(b)). On January 30, 2023, Twin A underwent conservative surgery (sectorial mastectomy plus sentinel lymph node dissection), finding a residual invasive carcinoma non-special type of 8 mm ypT1b, G3, ypN0 (0/3), TNBC Ki67 70%, tumor-infiltrating lymphocytes (TILs) 0%–10%, and non-evident lympho-vascular invasion.

As per multidisciplinary discussion, the patient was referred for adjuvant radiotherapy and continued Pembrolizumab 200 mg for 9 cycles in the adjuvant setting.

Twin B developed post-infusion fever after eight cycles of carboplatin and paclitaxel and two cycles of Pembrolizumab: hemoculture and urine culture were negative, antigen nasal swab for COVID-19 was repeatedly negative, and acute-phase proteins were within normal ranges. Due to the persistence of the fever, a transesophageal echocardiography was performed resulting in being negative for valvular vegetation.

Chemo-immunotherapy was carried out with frequent withdrawals due to fever, an episode of transaminitis G2, and diarrhea G1 (up to 4 liquid stools per day): the patient started methylprednisolone 1 mg/kg/die with no complete resolution of the side effects. The fourth cycle of EC and Pembrolizumab was administered regularly.

At the end of neoadjuvant chemotherapy, breast MRI showed a partial clinical response (Figure 3(b)).

In March 2023, the patient underwent surgery (sectorial left mastectomy plus sentinel lymph node dissection): the histologic report showed a pathologic complete response (pCR) in both breast and axilla, ypT0 ypN0 (0/4).

**Figure 4.**
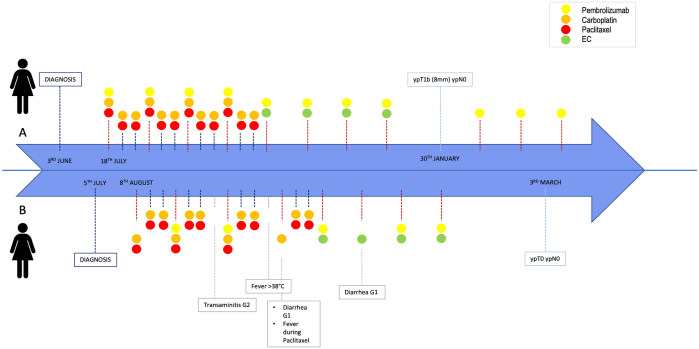
Timeline of twins’ clinical history.

The timeline of therapy administration until surgery is summarized in Figure 4.

After surgery, the patient developed abdominal pain, unintentional weight loss of 15 kg, worsening of diarrhea, and fatty stools with undigested food. Further examinations were requested (Table 1).

**Table 1.** Assessments requested for B’s malabsorption syndrome.

Test	Result	Comment
Fecal culture	Negative for parasitosis or infections	Negative
Fecal calprotectin dosage	393 μg/g	Altered
Pancreatic MRI	Negative	Negative
Lipase	5 U/L	Out of normal range (13–60 U/L)
Amylase	1 U/L	Out of normal range (5–53 U/L)
PTH	<1 ng/L	Out of normal range (12–88 ng/L)
Fecal elastase-1	8 μg/g	Out of normal range (200–1000 μg/g)

PTH, Parathyroid Hormone.

The examinations led to the diagnosis of pancreatic exocrine insufficiency, hypoparathyroidism, and colitis; a colonoscopy was performed showing immune-related microscopic colitis and an esophagogastric-duodenoscopy showed iatrogenic duodenitis and acute gastritis. The patient received pancreatic enzyme replacement therapy and corticosteroid therapy with prednisone 50 mg/die. The high-dose corticosteroid therapy led to iatrogenic osteoporosis with consequent pathological vertebral fracture that required vertebroplasty (July 2023).

The patient has permanently discontinued Pembrolizumab in the post-neoadjuvant setting.

## Outcome and follow-up

According to the most recent assessment, Twin A continues her daily activities and completed Pembrolizumab in the adjuvant setting. Twin B is tapering steroidal therapy; her levels of amylase and lipase remain below the limit, and she still requires enzymatic replacement treatment. Both twins are currently without evidence of recurrence.

## Discussion

This is an uncommon clinical situation involving two monozygotic twins diagnosed with TNBC of the left breast with lymph node involvement, emerging simultaneously and not correlated with any pathogenetic variants among the genes commonly associated with genetic syndromes.

Hereditary predisposition likely plays a role, though no pathogenic variants have yet been identified in the two probands. The deceased maternal grandfather was diagnosed with BC, a rare condition (incidence of 0.5%–1% of all BC diagnoses^18^) more frequently associated with *BRCA2* mutation (present in approximately 14% of cases of male BC^19^). However, the rest of the family tree was unremarkable. The WES performed on Twin B did not yield informative results concerning SNV or CNV among cancer susceptibility genes, which would have been crucial for developing a surveillance program. Notably, the test did not sequence the entire human genome but focused on variants in genes related to the specific phenotype (TNBC). Certain variant types may not be detected by NGS, including large duplications/deletions, structural chromosomal rearrangements, triplet repeat expansions, large indels, mutations associated with triallelic inheritance, variants in the mitochondrial genome, and epigenetic effects. Only germline variants are detected, excluding somatic variants or mosaicisms. Thus, the absence of disease-causing variants does not definitely rule out a genetic diagnosis in the proband.

In addition, the test was only conducted on twin B, as she pursued it independently and it was not part of our Institution’s routine protocol. The fact that only one twin was analyzed significantly limits the ability to generate comparative hypotheses, as differences at the epigenomic level could not be assessed.

In addition, this case highlights differences in the response to neoadjuvant immunotherapy: both twins underwent the same treatment for BC at the same stage but exhibited different toxicities and outcomes. The Twin B had a total of five cycles of Pembrolizumab in the neoadjuvant setting, with three withdrawn due to severe toxicity. By contrast, the first twin managed to complete all eight cycles of neoadjuvant Pembrolizumab. Despite the Keynote-522 protocol, the continuation of Pembrolizumab regardless of the pathologic response (with different benefits observed in patients achieving pCR or not) was permitted,^15,16^ we decided to permanently interrupt the treatment for Twin B due to the severe toxicity she reported.

Little is known about predictors of immunotherapy response in early BC, and efforts are underway to better understand the mechanisms associated with treatment response and resistance. In contrast with the metastatic setting, programmed death-ligand-1 (PD-L1) expression seemed to predict pCR in pre-treated early BC.^15,20^ TILs are known to be prognostic in TNBC and their potential value has been investigated in the context of immunotherapy strategies. In early BC, higher TIL levels (>50%) seem to be associated with better outcomes in patients treated only with locoregional therapies^21^ and also correlate with better responses to anti-PD-1 in early settings.^22–24^ Unfortunately, since TIL levels are not yet a recognized biomarker for orienting clinical decisions, we do not dispose of their evaluation in the histological report of the primary biopsy of our patients. However, twin A, who presented residual infiltrating component after neoadjuvant treatment, showed a low level of TILs, which might have affected her response to early treatment, consistent with the literature.^25^ Moreover, the fact that the other twin has achieved a pCR further limits the comparison possibilities after completing therapy in the neoadjuvant setting. The same applies to other biomarkers, also not currently implemented in clinical practice for prognostic or therapeutic purposes in this setting, such as PD-L1 expression, Tumor Proportion Score, and combined positive score, which we do not dispose of.

In the Keynote-522 trial, pCR confirmed being highly prognostic with respect to event-free survival (EFS)^26^ and overall survival^27^ in patients with early TNBC. However, the 5-year EFS of patients reaching pCR in the placebo arm was nearly 90%,^26^ suggesting the need to identify which patients are likely to have a favorable prognosis without immunotherapy, to reduce immune-related toxicity in these patients. Indeed, immune-related adverse events (irAEs) of any grade were reported in 35.0% of patients receiving pembrolizumab versus 13.1% of those receiving placebo, mostly related to thyroid dysfunctions.^27^ However, several real-world retrospective analyses revealed significative higher rates of irAEs (above 60% in patients exposed to pembrolizumab),^28,29^ maybe due to the less controlled environment of routine practice as compared to the highly regulated setting of a clinical trial.

Several studies have reported a positive correlation between the development of adverse events and the response to immunotherapy in the context of different tumors.^30,31^ However, few data are available for immunotherapy administration in the early setting and BC in particular. A recent retrospective real-world analysis of toxicity and efficacy of pembrolizumab for the treatment of early TNBC confirmed a statistically significant correlation with neoadjuvant and adjuvant irAEs and pCR, while early discontinuation of chemotherapy (before week 12) significantly correlated with a lower pCR.^29^ Taken together, those data emphasize the need for careful patient selection and monitoring during the administration of immunotherapy combinations.

Concerning the peculiar toxicity encountered, only anecdotical cases have been published about exocrine pancreatic insufficiency due to immunotherapy, all in the metastatic setting.^32–34^ There are no clear guidelines to treat such adverse events due to their rarity. We decided to implement corticosteroids to reduce pancreatic inflammation and interfere with the pathogenetic immune-mediated mechanism. The patient received replacement therapy with amylase and lipase enzymes with the support of multidisciplinary collaboration. However, the treatment itself was the cause of severe vertebral osteoporosis that requested surgical intervention, thus impacting the patient’s length of hospitalization and her quality of life long term.

## Conclusion

In conclusion, this exceptional case highlights the importance of increasing our knowledge of the pathogenesis of BC, including genetic and epigenetic features, to provide patients with adequate tailored therapeutic and risk-adapted follow-up strategies. Much is still under investigation in the field of immunotherapy, starting from the identification of biomarkers predicting response and toxicities. Factors such as the tumor microenvironment, immune system variability, and epigenetic differences may have contributed to the discrepancies we observed between these two patients.

Furthermore, when an immune-mediated adverse event arises, multidisciplinary management should be guaranteed. This approach ensures that the patient receives the finest supportive therapy, limiting both short- and long-term toxicities, and safeguarding their quality of life, especially when treated in early settings.

## Supplemental Material

sj-pdf-1-tam-10.1177_17588359241297565 – Supplemental material for Different activity and toxicity of immunotherapy in monozygotic twins diagnosed with early triple-negative breast cancer: a case report Supplemental material, sj-pdf-1-tam-10.1177_17588359241297565 for Different activity and toxicity of immunotherapy in monozygotic twins diagnosed with early triple-negative breast cancer: a case report by Marta Perachino, Lucia Del Mastro, Giulia Buzzatti, Lucia Trevisan, Maria Grazia Razeti, Andrea Bellodi, Stefano Spinaci, Davide Soldato, Matteo Lambertini and Francesca Poggio in Therapeutic Advances in Medical Oncology

## Declarations

**Ethics approval and consent to participate:** The present case report was approved by our local Ethics Committee.

**Consent for publication:** Patients were informed in writing and provided explicit consent for using their data to scientific purpose.

**Availability of data and materials:** Available upon reasonable request to the corresponding author.
